# Supplementary material for: A multi-scale pipeline linking drug transcriptomics with pharmacokinetics predicts in vivo interactions of tuberculosis drugs
Source: Sci Rep. 2021 Mar 11;11:5643. doi: 10.1038/s41598-021-84827-0 (PMC7971003; doi:10.1038/s41598-021-84827-0)
Supplement: Supplementary file 1 — Supplementary Information. [file 41598_2021_84827_MOESM1_ESM.pdf]

Supplementary Information

A multi-scale pipeline linking drug transcriptomics with pharmacokinetics predicts *in vivo* interactions of tuberculosis drugs

Joseph Cicchese[1], Awanti Sambarey[2], Denise Kirschner[3]*, Jennifer Linderman[1]*, Sriram Chandrasekaran[2]*

[1]Department of Chemical Engineering, University of Michigan, Ann Arbor, Michigan, USA
[2]Department of Biomedical Engineering, University of Michigan, Ann Arbor, Michigan, USA
[3]Department of Microbiology and Immunology, University of Michigan Medical School, Ann Arbor, Michigan, USA

Supplementary Table 1. List of regimens simulated and their corresponding fractional inhibitory concentrations (FIC). The antibiotics included and their abbreviations and standard doses are isoniazid (H, 5 mg/kg), rifampin (R, 10 mg/kg), ethambutol (E, 20 mg/kg), pyrazinamide (Z, 25 mg/kg), moxifloxacin (M, 7 mg/kg), and levofloxacin (L, 17 mg/kg). The regimen names give the single letter abbreviation, followed by the dose in mg/kg for that antibiotic. If no number is listed, the standard dose was used. The doses per week (dpw) are listed at the end of the regimen. If no doses per week is listed, doses were simulated as administered daily. The regimens used for validation of the model are labeled with an asterisk, corresponding to the regimens listed in Figure 4 and referenced from Bonnet *et al.* (2017)[38].

**Regimen**	**FIC**
HRZE	0.82
HR	1.35
HZ	0.97
HE	1.46
RZ	1.33
ZE	1.12
RE	0.74
HRZ	0.74
HRE	0.94
HZE	0.91
RZE	0.78
HMZE	1.05
RMZE	0.96
HM	2.01
RM	2.31
MZ	1.99
ME	1.8
HRM	1.08
HMZ	1.1
HME	1.19
RMZ	1.33
RME	0.99
MZE	1.19
HRMZ	0.92
HRME	0.93
HL	1.25
RL	1.53
LZ	1.47
LE	1.28
HRL	1.01
HLZ	1.11
HLE	1.15

**Regimen**	**FIC**
RLZ	1.22
RLE	1.12
LZE	1.28
HRLZ	0.8
HRLE	0.89
HLZE	1.01
RLZE	0.94
H6E15*	1.46
H6E25*	1.46
R11E25*	0.74
R23.5E25*	0.74
H11R10*	1.35
H6R10E16*	0.94
H6R10E25*	0.94
H10R10E25*	0.94
R24Z64E90dpw1*	0.78
H16R12Z49dpw2*	0.74
H6R10Z27E16*	0.82
H5R10M7Z25dpw5*	0.92
R10M7Z25E15*	0.96
H14R10M9Z34dpw3*	0.92
H6E6*	1.46
H10E25*	1.46
R23.5E45dpw2*	0.74
R23.5E90dpw1*	0.74
H6R6E10*	0.94
H15R10E40dpw2*	0.94
H5R10Z25E17.5*	0.82
H6R9Z24E16dpw3*	0.82
H14R13Z47E29dpw3*	0.82

*Used for validation

Supplementary Table 2 Host immune parameters used with *GranSim* to generate the granuloma bio-repository. Timestep units represent 10-minute time steps in the agent-based simulation. The minimum and maximum values for parameter ranges used in sampling are listed. Parameters are based on previous *GranSim* studies[24,33].

Parameter Definition	Units	Min	Max
# immune cell deaths causing compartment caseation		6	10
Time to heal caseated compartment	Timesteps	909	1365
TNF threshold for causing immune cell apoptosis	Molecules	690	1035
Rate constant for TNF-induced apoptosis	1/s	1.36e-6	2.04e-6
Minimum chemokine concentration to induce chemotaxis	Molecules	0.27	0.41
Maximum chemokine concentration to induce chemotaxis	Molecules	392	588
Initial density of macrophages	Fraction of grid compartments	0.019	0.029
Time between resting macrophage movements	Timesteps	4	6
Time between active macrophage movements	Timesteps	15	23
Time between infected macrophage movements	Timesteps	169	255
TNF threshold to induce NFkB activation	Molecules	42.8	64.1
Rate constant for NFkB activation	1/s	6.77e-6	1.01e-5
Probability resting macrophage kills extracellular Mtb		0.0738	0.111
Killing probability adjustment for resting macrophages with NFkB activation		0.129	0.194

# bacteria to cause NFkB activation		236	354
# bacteria for macrophage to become chronically infected		12	18
# bacteria to cause macrophage to burst		19	29
# bacteria activated macrophage can phagocytose		3	5
Probability activated macrophage will will heal a caseated compartment		0.00459	0.00687
Probability a T-cell will move to same compartment as a macrophage		0.0367	0.0550
Probability IFNγ producing T-cell induces Fas/FasL apoptosis		0.0293	0.0439
Probability IFNγ producing T-cell also produces TNF		0.0514	0.0770
Probability cytotoxic T-cell kills macrophage		0.00505	0.0121
Probability cytotoxic T-cell kills a macrophage and all its intracellular bacteria		0.619	0.928
Probability regulatory T-cell deactivates macrophage		0.00584	0.00876
Time when T-cell recruitment begins	Timesteps	3225	4722
Time delay after T-cell recruitment begins until maximal recruitment rate	Timesteps	650	976
Macrophage maximal recruitment probability		0.0241	0.0361
Macrophage threshold for recruitment by chemokines	Molecules	0.641	0.960
Macrophage threshold for recruitment by TNF	Molecules	0.00859	0.0129

Macrophage half saturation for recruitment by TNF	Molecules	1.22	1.82
Macrophage half saturation for recruitment by chemokine	Molecules	1.68	2.52
IFNγ producing T-cell maximal recruitment probability		0.0484	0.0726
IFNγ producing T-cell threshold for recruitment by chemokine	Molecules	0.0535	0.0802
IFNγ producing T-cell threshold for recruitment by TNF	Molecules	1.01	1.51
IFNγ producing T-cell half saturation for recruitment by TNF	Molecules	1.22	1.82
IFNγ producing T-cell half saturation for recruitment by chemokine	Molecules	1.64	2.46
Probability a IFNγ producing T-cell is cognate		0.0437	0.0655
Cytotoxic T-cell maximal recruitment probability		0.0370	0.0554
Cytotoxic T-cell threshold for recruitment by chemokine	Molecules	3.55	5.32
Cytotoxic T-cell threshold for recruitment by TNF	Molecules	0.920	1.38
Cytotoxic T-cell half saturation for recruitment by TNF	Molecules	0.715	1.07
Cytotoxic T-cell half saturation for recruitment by chemokine	Molecules	5.24	7.86
Probability a cytotoxic T-cell is cognate		0.0414	0.0620
Regulatory T-cell maximal recruitment probability		0.0246	0.0369
Regulatory T-cell threshold for recruitment by chemokine	Molecules	2.03	3.04

Regulatory T-cell threshold for recruitment by TNF	Molecules	1.65	2.47
Regulatory T-cell half saturation for recruitment by TNF	Molecules	2.00	3.00
Regulatory T-cell half saturation for recruitment by chemokine	Molecules	1.23	1.84
Probability a regulatory T-cell is cognate		0.0400	0.0600

Supplementary Table 3. For four regimens (HRZE, RE, HE and RM), the table shows the PRCC values relating the plasma PK parameters to the predicted iDIS for non-replicating Mtb during the first dose of treatment. All PRCC values reported are significant with p<0.01 and NS designates that the parameter was not significant. The parameters for each antibiotic listed include the absorption rate constant (kAbs), the intercompartmental clearance (Q), the volume of distribution for the plasma (central) compartment (Vol. Dist. Cent.), the volume of distribution for the peripheral compartment (Vol. Dist. Periph.), and the clearance rate constant (CL).

	INH					RIF					EMB					PZA					MXF				
	kAbs	Q	Vol. Dist. Cent	Vol. Dist. Periph	CL	kAbs	Q	Vol. Dist. Cent	Vol. Dist. Periph	CL	kAbs	Q	Vol. Dist. Cent	Vol. Dist. Periph	CL	kAbs	Q	Vol. Dist. Cent	Vol. Dist. Periph	CL	kAbs	Q	Vol. Dist. Cent	Vol. Dist. Periph	CL
**HRZE**	NS	-0.66	0.34	-0.18	-0.20	0.28	NS	NS	NS	0.89	0.43	NS	-0.26	NS	-0.56	NS	NS	0.54	NS	0.21					
**RE**						NS	NS	NS	NS	0.90	0.74	-0.27	-0.23	-0.20	-0.92										
**HE**	-0.31	-0.87	0.67	-0.33	-0.39						-0.68	0.26	0.30	0.20	0.88										
**RM**						NS	NS	NS	-0.26	-0.83											NS	-0.12	0.19	0.95	0.99

Supplementary Table 4. For four regimens (HRZE, RE, HE and RM), the table shows the PRCC values relating the tissue PK parameters to the predicted iDIS for non-replicating Mtb during the first dose of treatment. All PRCC values reported are significant with p<0.01 and NS designates that the parameter was not significant. The parameters for each antibiotic listed include the extracellular degradation rate constant (Deg Rate), the intracellular degradation rate constant (Int Deg Rate), the effective diffusivity (Diff), the cellular uptake ratio (Cell Up.) and the vascular permeability (Perm.). Degradation rate constants for MXF are not included because those rate constants are set to zero in the simulation.

	INH					RIF					EMB					PZA					MXF		
	Deg Rate	Int Deg Rate	Diff	Cell Up.	Perm.	Deg Rate	Int Deg Rate	Diff	Cell Up.	Perm.	Deg Rate	Int Deg Rate	Diff	Cell Up.	Perm.	Deg Rate	Int Deg Rate	Diff	Cell Up	Perm.	Diff	Cell Up.	Perm
**HRZE**	NS	NS	NS	NS	NS	NS	0.76	0.10	0.72	-0.74	NS	-0.11	0.26	-0.45	0.56	NS	0.12	NS	0.23	NS			
**RE**						NS	0.66	NS	0.64	-0.62	NS	-0.27	0.50	-0.70	0.84								
**HE**	NS	NS	-0.10	NS	-0.25						-0.10	0.36	-0.61	0.75	-0.87								
**RM**						NS	-0.27	NS	-0.30	0.23											-0.27	0.32	-0.42

Supplementary Table 5. Plasma pharmacokinetic parameters listed with the ranges (min, max) for the antibiotics included in the regimen combinations: isoniazid (INH), rifampin (RIF), ethambutol (EMB), pyrazinamide (PZA), moxifloxacin (MXF) and levofloxacin (LVX). Parameters values are based on the results of calibration efforts in previously published studies[31,33].

**Parameter**	**Units**	**INH**	**RIF**	**EMB**	**PZA**	**MXF**	**LVX**
Absorption rate constant	1/h	0.50, 6.0	0.40, 2.5	0.10, 0.80	0.55, 0.75	2.0, 2.5	3.1, 9.4
Intercomp. clearance rate constant	L/(h*kg)	0.20, 0.70	2.0, 5.9	0.45, 0.70	0.10, 0.70	2.0, 16	0.14, 1.5
Central compartment volume of distribution	L/kg	0.50, 3.0	0.18, 0.57	0.80, 1.95	0.25, 0.75	0.33, 0.37	0.54, 1.1
Peripheral compartment volume of distribution	L/kg	25, 40	0.32, 0.97	8.1, 12.7	0.010, 0.050	0.5, 1.03	0.23, 0.69
Plasma clearance rate constant	L/(h*kg)	0.0080, 0.070	0.050, 0.30	0.3, 1.0	0.010, 0.050	0.074, 0.175	0.10, 0.14

Supplementary Table 6. The calibrated tissue pharmacokinetic parameters for the antibiotics included in the regimen combinations, isoniazid (INH), rifampin (RIF), ethambutol (EMB), pyrazinamide (PZA), moxifloxacin (MXF) and levofloxacin (LVX), based on previously published calibration studies[31,33].

**Parameter**	**INH**	**RIF**	**EMB**	**PZA**	**MXF**	**LVX**
Extracellular degradation rate constant (1/s)	6.94e-8	3.90e-8	1.73e-8	1.34e-8	0.0	0.0
Intraceullar degradation rate constant (1/s)	2.84e-6	2.59e-4	8.75e-6	2.26e-3	0.0	0.0
Effective diffusivity (cm^2/s)	6.58e-7	5.08e-8	5.20e-7	3.24e-6	1.4e-7	1.3e-6
Cellular accumulation ratio	1.13	24	5.95	0.593	7.00	3.81
Vascular permeability (cm/s)	1.34e-6	2.65e-7	1.33e-7	8.62e-6	3.0e-6	2.0e-6
Permeability coefficient	0.25	3.3	7.4	1	4.87	1.56
Fraction unbound to caseum	1	0.052	0.35	1	0.26	0.36

Supplementary Table 7. Pharmacodynamic parameters for each of the antibiotics: isoniazid (INH), rifampin (RIF), ethambutol (EMB), pyrazinamide (PZA), moxifloxacin (MXF) and levofloxacin (LVX). Parameters were estimated in calibrations from previously published studies[31,33]. Units of 1/timestep represent per model timestep of 10 minutes.

**Parameter**	**INH**	**RIF**	**EMB**	**PZA**	**MXF**	**LVX**
Intracellular C_{50} (mg/L)	0.070	20	5.22	70	28	34
Extracellular, replicating C_{50} (mg/L)	0.015	1.23	0.05	370	0.06	0.23
Extracellular, Non-replicating C_{50} (mg/L)	17.7	81	1000	370	30	30
Intracellular Emax (1/timestep)	0.0056	0.014	0.026	0.0006	0.01	0.01
Extracellular Emax (1/timestep)	0.0056	0.019	0.025	0.007	0.003	0.004
Intracellular hill constant, h	1	0.5	2.5	3.2	1.2	5.02
Extracellular hill constant, h	1	0.5	1.5	1	4.99	5.02

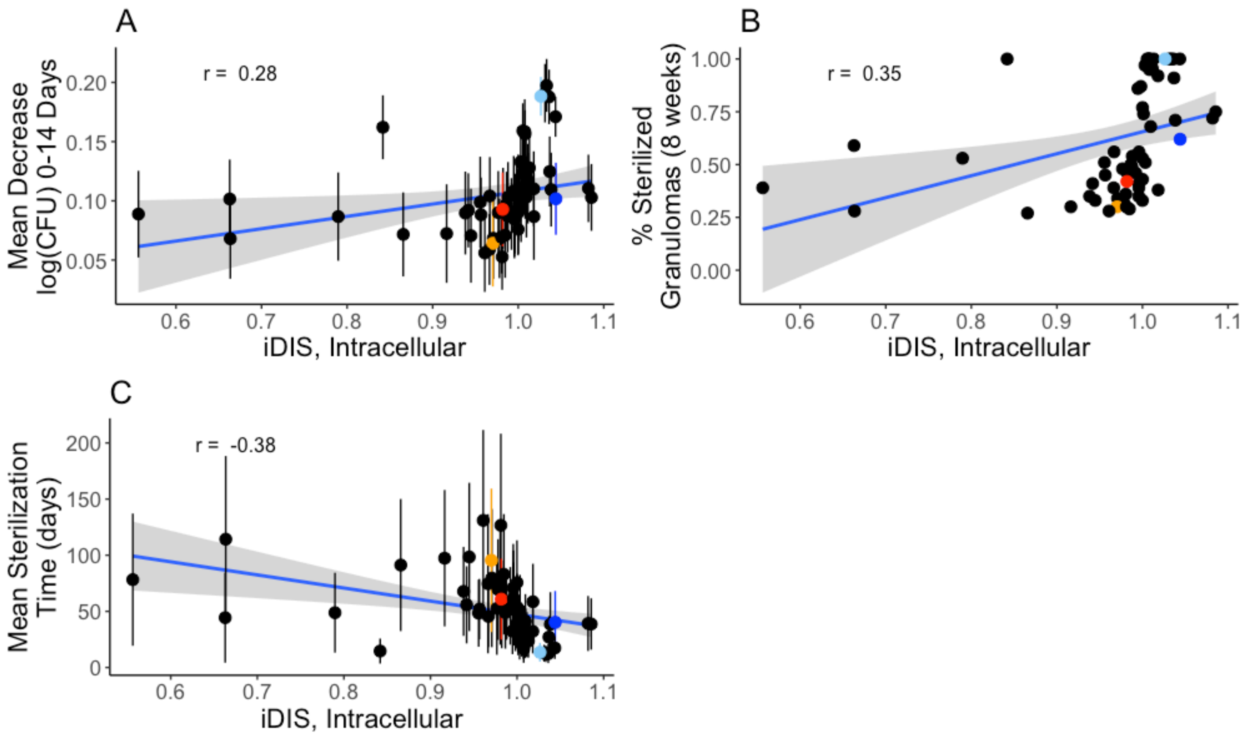

Supplementary Figure 1 Measures of regimen efficacy are correlated with interaction strength associated with intracellular replicating Mtb killing rate for 64 regimens. The mean decrease in log CFU (0-14) averaged over each 100 granulomas simulated for each regimen (A) and percentage of sterilized (negative) granulomas after eight weeks of treatment (B) are weakly positively correlated with the average interaction strength experienced by non-replicating Mtb during the first 24 hours of treatment with correlation coefficients of 0.28 and 0.35 respectively. Mean sterilization time for each regimen over 100 granulomas (C) is negatively correlated with the average interaction strength with a correlation coefficient of -0.38. Each point represents the regimen outcome measurement for a given regimen and error bars indicate +/- standard deviation from the sample of 100 granulomas simulated. The colored points correspond to the regimens HRZE (light blue), RE (dark blue), RM (red) and HE (orange).

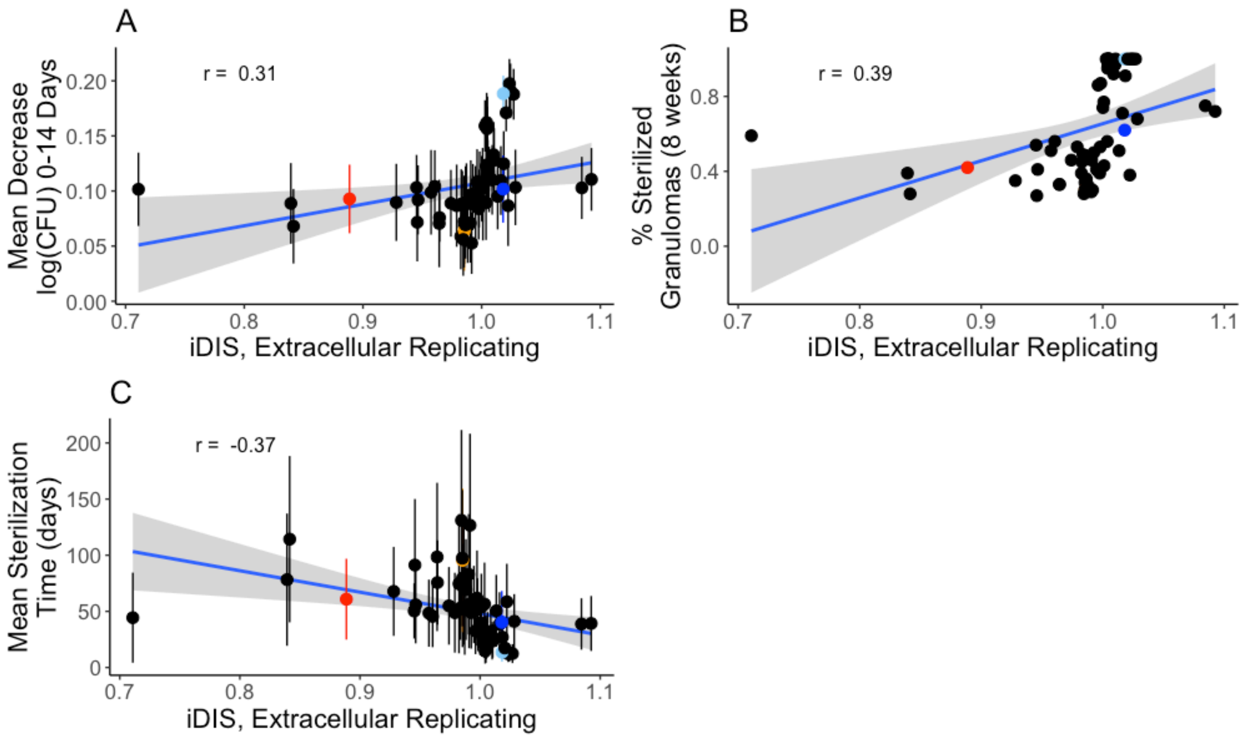

Supplementary Figure 2 Measures of regimen efficacy are correlated with interaction strength associated with extracellular replicating Mtb killing rate for 64 regimens. The mean decrease in log CFU (0-14) averaged over each 100 granulomas simulated for each regimen (A) and percentage of sterilized (negative) granulomas after eight weeks of treatment (B) are weakly positively correlated with the average interaction strength experienced by non-replicating Mtb during the first 24 hours of treatment with correlation coefficients of 0.28 and 0.35 respectively. Mean sterilization time for each regimen over 100 granulomas (C) is negatively correlated with the average interaction strength with a correlation coefficient of -0.38. Each point represents the regimen outcome measurement for a given regimen and error bars indicate +/- standard deviation from the sample of 100 granulomas simulated. The colored points correspond to the regimens HRZE (light blue), RE (dark blue), RM (red) and HE (orange).

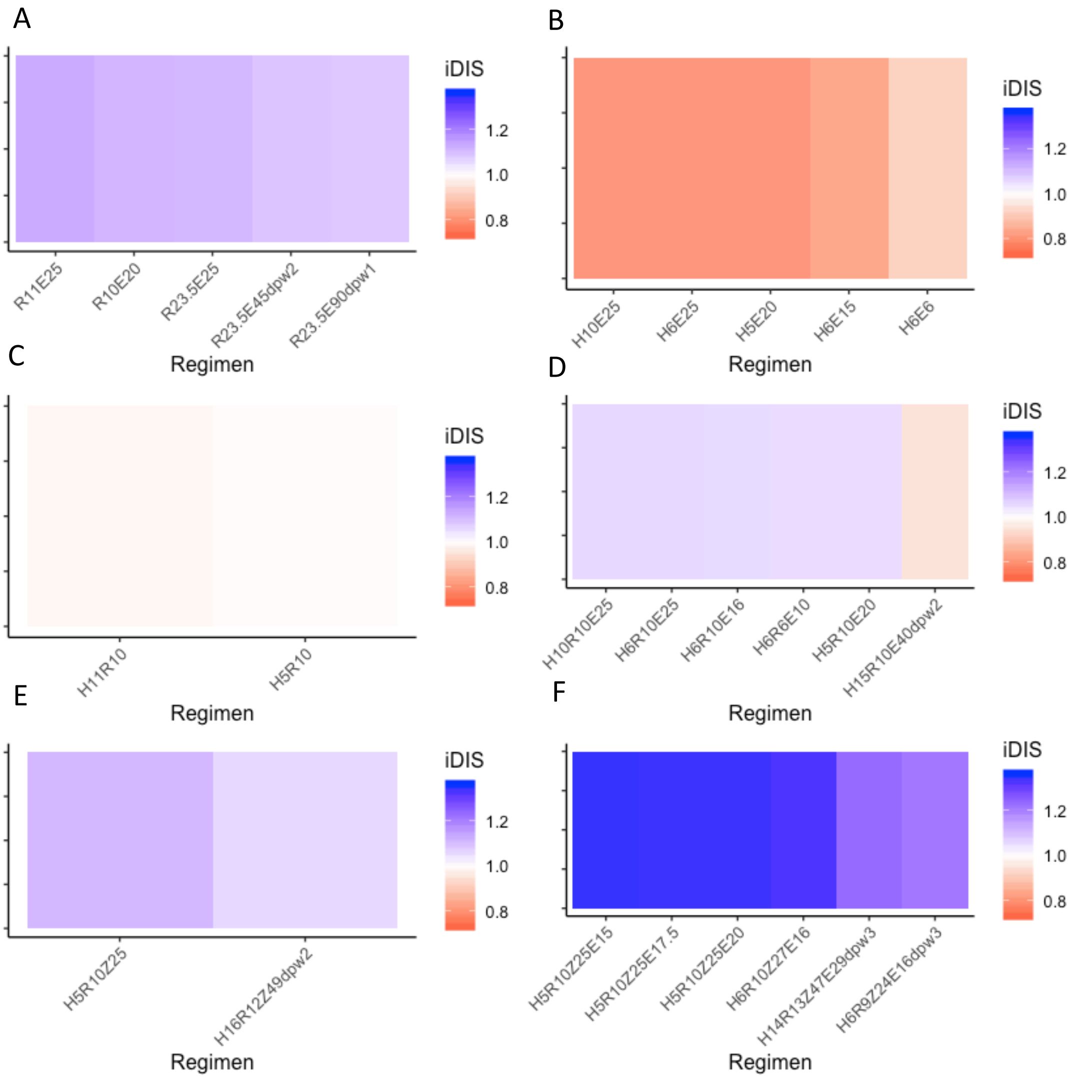

Supplementary Figure 3 Heat map of predicted iDIS value for different regimens of the same antibiotic combination. The list of regimens is ordered by decreasing predicted in vivo DIS for regimens involving the antibiotic combination RE (A), HE (B), HR (C), HRE (D), HRZ (E) and HRZE (F). For predicated IDIS blue represents synergy, white represents additivity, and red represents antagonism.
